# Angiotensin II and Angiotensin-(1-7) in Paraventricular Nucleus Modulate Cardiac Sympathetic Afferent Reflex in Renovascular Hypertensive Rats

**DOI:** 10.1371/journal.pone.0052557

**Published:** 2012-12-20

**Authors:** Hai-Jian Sun, Peng Li, Wei-Wei Chen, Xiao-Qing Xiong, Ying Han

**Affiliations:** Department of Physiology, Nanjing Medical University, Nanjing, Jiangsu, China; Max-Delbrück Center for Molecular Medicine (MDC), Germany

## Abstract

**Background:**

The enhanced cardiac sympathetic afferent reflex (CSAR) is involved in the sympathetic activation that contributes to the pathogenesis and progression of hypertension. Activation of AT_1_ receptors by angiotension (Ang) II in the paraventricular nucleus (PVN) augments the enhanced CSAR and sympathetic outflow in hypertension. The present study is designed to determine whether Ang-(1-7) in PVN plays the similar roles as Ang II and the interaction between Ang-(1-7) and Ang II on CSAR in renovascular hypertension.

**Methodology/Principal Findings:**

The two-kidney, one-clip (2K1C) method was used to induce renovascular hypertension. The CSAR was evaluated by the renal sympathetic nerve activity (RSNA) and mean arterial pressure (MAP) responses to epicardial application of capsaicin in sinoaortic-denervated and cervical-vagotomized rats with urethane and α-chloralose anesthesia. Either Ang II or Ang-(1-7) in PVN caused greater increases in RSNA and MAP, and enhancement in CSAR in 2K1C rats than in sham-operated (Sham) rats. Mas receptor antagonist A-779 and AT_1_ receptor antagonist losartan induced opposite effects to Ang-(1-7) or Ang II respectively in 2K1C rats, but losartan had no effects in Sham rats. Losartan but not the A-779 abolished the effects of Ang II, while A-779 but not the losartan blocked the effects of Ang-(1-7). PVN pretreatment with Ang-(1-7) dose-dependently augmented the RSNA, MAP, and CSAR responses to the Ang II in 2K1C rats. Ang II level, AT_1_ receptor and Mas receptor protein expression in PVN increased in 2K1C rats compared with Sham rats but Ang-(1-7) level did not.

**Conclusions:**

Ang-(1-7) in PVN is as effective as Ang II in enhancing the CSAR and increasing sympathetic outflow and both endogenous Ang-(1-7) and Ang II in PVN contribute to the enhanced CSAR and sympathetic outflow in renovascular hypertension. Ang-(1-7) in PVN potentiates the effects of Ang II in renovascular hypertension.

## Introduction

Cardiac sympathetic afferent reflex (CSAR) is known to be a positive-feedback, sympathoexcitatory cardiovascular reflex [1], [2]. Our previous studies have shown that the CSAR is enhanced in renovascular hypertensive rats [3], [4] which contributes to the excessive sympathetic activity and hypertension [5], [6]. Excessive sympathetic activity propels the pathogenesis of hypertension and progression of organ damage [7]–[9]. Intervention of the enhanced CSAR and sympathetic activity is considered to be an antihypertensive strategy [10]–[12].

It is well known that the paraventricular nucleus (PVN) is an important component of the central neurocircuitry of the CSAR [13] and plays a primary role in the integration of sympathetic output and cardiovascular activity via projections to the intermediolateral column of the spinal cord and the rostral ventrolateral medulla (RVLM) [14]. Ang II and Ang-(1-7) are now known as two important biological active peptides of renin–angiotensin system (RAS) family. Ang-(1-7) is formed either directly from Ang II or indirectly from Ang I through angiotensin converting enzyme 2 (ACE2). Many of Ang-(1-7) effects are primarily mediated by Mas receptors [15] and are selectively blocked by its specific antagonist D-Alanine-Ang-(1-7) (A-779) [16]. While most effects of Ang II are mediated by the AT_1_ receptors and blocked by AT_1_ receptor antagonist losartan [17]. The endogenous Ang-(1-7) level in the hypothalamus of rats is comparable to Ang I and Ang II [18]. Ang-(1-7) immunoreactive staining and Mas receptor protein expression and immunofluorescence are found in the PVN [19]–[21]. AT_1_ receptors are also densely distributed in the PVN [22], [23].

It has been found that Ang-(1-7) shows the opposite effects to Ang II on peripheral tissues [24], [25]. However our recent study has shown that microinjection of either Ang-(1-7) or Ang II into the RVLM enhances the CSAR and increases renal sympathetic nerve activity (RSNA) and mean arterial pressure (MAP) in normal rats [26]. Pretreatment with Ang-(1-7) before Ang II enhances Ang II induced phosphorylation of ERK1/2 in mouse bone marrow-derived dendritic cells [27]. Chronic infusion of both A-779 and losartan into the PVN prevent hypertension in a rat model of sleep apnea [28]. We found that the activation of AT_1_ receptors by Ang II in the PVN augments the enhanced CSAR and sympathetic outflow in both chronic heart failure (CHF) rats [29], [30] and renovascular hypertensive rats [6], suggesting that the activity of Ang II and AT_1_ receptors in PVN is involved in modulating the enhanced CSAR and sympathetic outflow in these cardiovascular diseases. Study also indicates that the blockade of endogenous Ang-(1-7) by microinjection of A-779 into the PVN reduces renal sympathetic tone in rats [31]. However whether Ang-(1-7) in PVN plays the similar roles as Ang II in modulating the CSAR, sympathetic outflow and blood pressure and the interaction between Ang-(1-7) and Ang II in renovascular hypertension is still unclear. The present study was designed to determine whether Ang-(1-7) in PVN is as active as Ang II in these effects and to indicate the interaction between Ang-(1-7) and Ang II in renovascular hypertension.

## Materials and Methods

Experiments were carried out in male Sprague–Dawley rats. The procedures were approved by the Experimental Animal Care and Use Committee of Nanjing Medical University (No. 20110523) and complied with the Guide for the Care and Use of Laboratory Animals (NIH publication no. 85–23, revised 1996). The rats were kept in a temperature-controlled room on a 12 h–12 h light–dark cycle with free access to standard chow and tap water.

### Renovascular Hypertensive Model

Renovascular hypertension was induced by Goldblatt two-kidney one-clip (2K1C) method as our previous reports [3]–[5]. To minimize the stress-induced systolic blood pressure (SBP) fluctuation, the rat was trained by measuring SBP daily for at least 10 days before 2K1C or sham operation. Then a retroperitoneal flank incision was performed in rat weighing 160–180 g under anaesthesia with intraperitoneal administration of sodium pentobarbital (50 mg kg^-1^). The right renal artery was exposed and partly occluded by placing a U-shaped silver clip with an internal diameter of 0.20 mm on the artery to induce renovascular hypertension. Normotensive sham-operated (Sham) rat received similar surgical process except using silver clip. Acute experiments were carried out at the end of the 4th week after the surgery. The criterion of hypertension in the present study is set as SBP>160 mm Hg [3]–[5]. Only rats with SBP>160 mm Hg underwent acute experiments in 2K1C rats. Seven rats were excluded since the SBP in these rats was not high enough to meet the criterion in 2K1C rats mentioned above.

### SBP Measurements

The SBP of tail artery was measured weekly throughout the 4 weeks period in conscious rat by using a noninvasive computerized tail-cuff system (NIBP, ADInstruments, Australia) [3]–[5]. The rats were warmed for 10–20 min at 28°C before the measurements in order to allow the detection of tail artery pulsations and to achieve the steady pulse level. The SBP was obtained by averaging 10 measurements.

### General Procedures of Acute Experiment

At the end of the 4th week after 2K1C or sham operation, each rat was intraperitoneally anesthetized with urethane (800 mg kg^−1^) and α-chloralose (40 mg kg^−1^). Supplemental doses of anesthesia were used to maintain an appropriate level of anaesthesia that was assessed by the absence of corneal reﬂexes and paw withdrawal response to a noxious pinch. The rat was mechanical ventilated with room air using a rodent ventilator (model 683, Harved Apparatus Inc, USA). The right carotid artery was cannulated and connected with a pressure transducer (MLT0380, ADInstruments, Australia) for continuous recording of arterial blood pressure (ABP), MAP and heart rate (HR).

### Vagotomy and Baroreceptor Denervation

Vagotomy and baroreceptor denervation were carried out to minimize the confounding effect of the baroreflex on sympathetic activity and blood pressure [4], [6], [32]. The bilateral vagi and carotid sinus nerves were identified, tied and sectioned in the neck. All other nerve fibers that were visible in carotid sinus areas were also cut. The common carotid arteries and carotid bifurcation were stripped of adventitial tissues from 4 mm below the bifurcation to 4 mm above. The vessels were painted with 10% phenol solution to destroy any remaining nerve fibers in this area. The effectiveness of baroreceptor denervation was identified by the criterion that the HR change is less than 5 beats min^-1^ after intravenous injection of phenylephrine (20 µg kg^−1^) that induced an increase in MAP between 25 and 40 mm Hg.

### RSNA Recordings

A retroperitoneal incision was made and the left renal sympathetic nerve was isolated. The renal nerve was cut distally to eliminate its afferent activity. The nerve was placed on a pair of silver electrodes and immersed in warm mineral oil. The nerve signals were amplified with a four channel AC/DC differential amplifier (DP-304, Warner Instruments, Hamden, CT, USA) with a high pass filter at 10 Hz and a low pass filter at 3,000 Hz. The RSNA was integrated at a time constant of 100 ms. At the end of each experiment, the background noise was determined after section of the central end of the nerve [26] and was subtracted from the integrated values of the RSNA. The raw and integrated RSNA, ABP, MAP and HR were simultaneously recorded with a PowerLab data acquisition system (8SP, ADInstruments, Australia).

### Evaluation of CSAR

A limited left lateral thoracotomy was performed and the heart was exposed, the pericardium was removed. The CSAR was elicited by epicardial application of a piece of filter paper (3 mm × 3 mm) containing capsaicin (1.0 nmol in 2.0 µl) on the anterior wall of the left ventricle which stimulated cardiac sympathetic afferent nerves. One minute later, the filter paper was removed and the ventricular surface was rinsed three times with 10 ml of normal saline (38°C). The CSAR was evaluated by the RSNA and MAP responses to the epicardial application of capsaicin [32], [33].

### PVN Microinjection

The rats were placed in a stereotaxic frame (Stoelting, Chicago, USA). The stereotaxic coordinates for the PVN are 1.8 mm caudal from bregma, 0.4 mm lateral to the midline and 7.9 mm ventral to the dorsal surface according to Paxinos & Watson’s rat atlas. The bilateral PVN microinjections were completed within 1 min and the microinjection volume was 50 nL for each side of the PVN. At the end of the experiment, 50 nL of Evans Blue dye (2%) was injected into each microinjection site. The microinjection sites were histologically verified with microscope. Rats with microinjection sites outside the PVN were excluded from data analysis.

### Measurement of Ang II and Ang-(1-7) Levels

PVN sample tissues were collected and homogenized as previously reported [3], [34]. The total protein in the homogenate supernatant was extracted and measured by using protein assay kit (BCA; Pierce). The level of Ang II in PVN tissue homogenate supernatant was measured using an enzyme-linked immunoassay kit (USCN Life Science Inc., USA). The Ang-(1-7) level in PVN was determined by a commercial peptide enzyme immunoassay kit (MyBioSource LLC, USA) [35]. For both assays, the manufacturer’s instructions were followed.

### Measurement of AT_1_ Receptor and Mas Receptor Protein Expression

The AT_1_ receptor and Mas receptor protein expression in the PVN were determined with Western blotting method as previous reports [21], [34], [36]. Briefly, proteins in PVN tissue homogenate supernatant were separated on a 10% SDS–PAGE and transferred to a nitrocellulose membrane. Membrane was then probed with rabbit polyclonal antibody against AT_1_ receptors (1∶300, Santa Cruz Biotechnology, Santa Cruz, CA, USA) or Mas receptors antibody (1∶200, Alomone Labs, Israel). This was followed by incubation with horseradish peroxidase–conjugated goat anti-rabbit IgG (1∶5000; Immunology Consultants Lab, USA). The bands were visualized by enhanced chemiluminescence using the ECL system (Pierce Chemical). GAPDH (Bioworld Technology Inc., USA) protein was used as loading control. The total amount of AT_1_ receptors protein or Mas receptors protein is expressed as the percentage of AT_1_ receptors or Mas receptors to GAPDH protein.

### Chemicals

Angiotensin-(1-7) and D-Alanine-Ang-(1-7) (A-779) were purchased from Bachem (Bubendorf, Switzerland). Angiotensin II and capsaicin were purchased from Sigma Chemical Co (St. Louis, MO, USA). Losartan was a gift from Merck. All the chemicals were dissolved in normal saline.

### Protocols

#### Protocol 1

The PVN microinjection of saline, Ang II (0.3 nmol), three doses of Ang-(1-7) (0.03, 0.3 and 3 nmol), and Ang II (0.3 nmol) combined with Ang-(1-7) (0.3 nmol) on the baseline RSNA and MAP and CSAR were carried out in six groups of 2K1C rats and six groups of Sham rats, respectively (n = 6 for each group). For the Ang II combined with Ang-(1-7) group, Ang-(1-7) and Ang II were administered simultaneously. CSAR was evaluated by the RSNA and MAP responses to epicardial application of capsaicin (1 nmol) at the fifth minute after the PVN microinjection of Ang II or Ang-(1-7). The RSNA and MAP changes caused by the PVN microinjection were determined by averaging 2 min of the maximal responses. The RSNA and MAP responses to capsaicin were determined beginning at the 15th sec after epicardial application of capsaicin by averaging 30 sec of the parameters.

#### Protocol 2

The effects of PVN pretreatment with saline, three doses of Ang-(1-7) (0.03, 0.3 and 3 nmol) on the baseline RSNA and MAP and CSAR responses to the Ang II were determined in four groups of 2K1C rats and four groups of Sham rats respectively (n = 6 for each group). The PVN pretreatment with Ang-(1-7) was administered five min before Ang II. The CSAR was evaluated at the fifth minute after the PVN microinjection of Ang II.

#### Protocol 3

The PVN microinjection of saline, AT_1_ receptor antagonist losartan (50 nmol), Mas receptor antagonist A-779 (3 nmol) as well as Ang II (0.3 nmol) or Ang-(1-7) (0.3 nmol) pretreated with losartan and Ang II or Ang-(1-7) pretreated with A-779 on the baseline RSNA, MAP and CSAR were carried out in seven groups of 2K1C rats and seven groups of Sham rats, respectively (n = 6 for each group). CSAR was evaluated at the eighth minute after the PVN microinjection of A-779 or losartan. For the pretreated groups, A-779 or losartan was administered eight min before Ang-(1-7) or Ang II, CSAR was evaluated at the fifth minute after Ang II or Ang-(1-7).

#### Protocol 4

Ang II and Ang-(1-7) levels in the PVN were determined in 5 Sham rats and 5 2K1C rats. Furthermore, AT_1_ receptor and Mas receptor protein expression in the PVN were determined in other 5 Sham rats and 5 2K1C rats.

### Statistical Analysis

Comparisons between two observations in the same animal were assessed by Student’s paired *t* test. One-way or two-way ANOVA was used followed by Bonferroni test for post hoc analysis when multiple comparisons were made. All data were expressed as mean ± SE. *P*<0.05 was considered statistically significant.

## Results

### General Data

At the end of the 4th week after 2K1C or sham operation, there were no significant difference in body weight and baseline HR between Sham rats and 2K1C rats. But both SBP of tail artery in conscious state and MAP of carotid artery under anesthesia in 2K1C rats were significantly higher than those in Sham rats (Table 1). There was no significant difference in the SBP and MAP among any subgroups within Sham rats or within 2K1C rats.

**Table 1:** The body weight, SBP, baseline MAP and baseline HR at the end of the fourth week.

	Sham	2K1C
n	112	112
Body Weight, g	329.6±1.7	324.3±1.5
SBP, mm Hg	120.8±1.0	194.3±2.0*
Baseline MAP, mm Hg	90.6±1.1	136.4±1.5*
Baseline HR, beats/min	352.9±4.1	360.7±4.2

The systolic blood pressure (SBP) of tail artery was measured without anesthesia by use of a noninvasive computerized tail-cuff system. The baseline mean arterial pressure (MAP) and heart rate (HR) were measured under anesthesia with a pressure transducer through a catheter placed in the right carotid artery. Values are expressed as mean ± SE.

* P<0.05 compared with the Sham.

### Effects of Ang II and Ang-(1-7)

**Figure 1:**
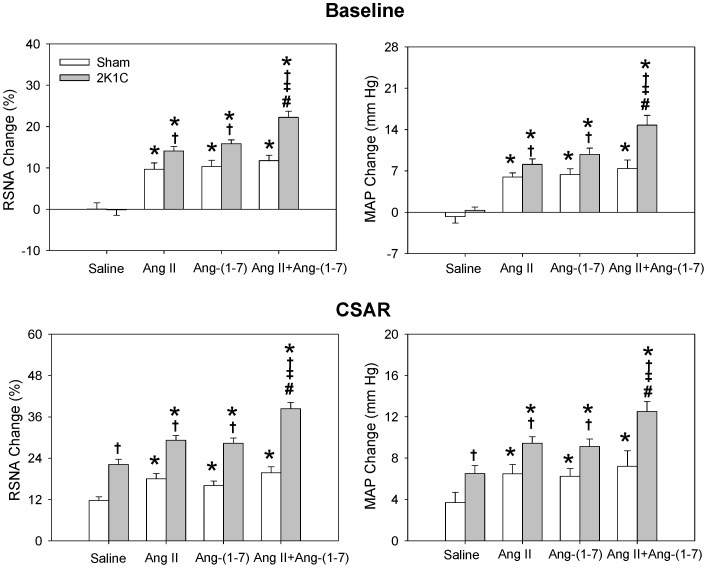
Effects of PVN microinjection of saline, Ang II (0.3 nmol), Ang-(1-7) (0.3 nmol) and Ang II+Ang-(1-7) on the baseline RSNA and MAP and CSAR. The CSAR was evaluated by the RSNA and MAP responses to epicardial application of capsaicin (1 nmol). Values are mean ± SE. * P<0.05 compared with saline. † P<0.05 compared with Sham. ‡ P<0.05 compared with Ang II alone. # P<0.05 compared with Ang-(1-7) alone. n = 6 for each group.

**Figure 2:**
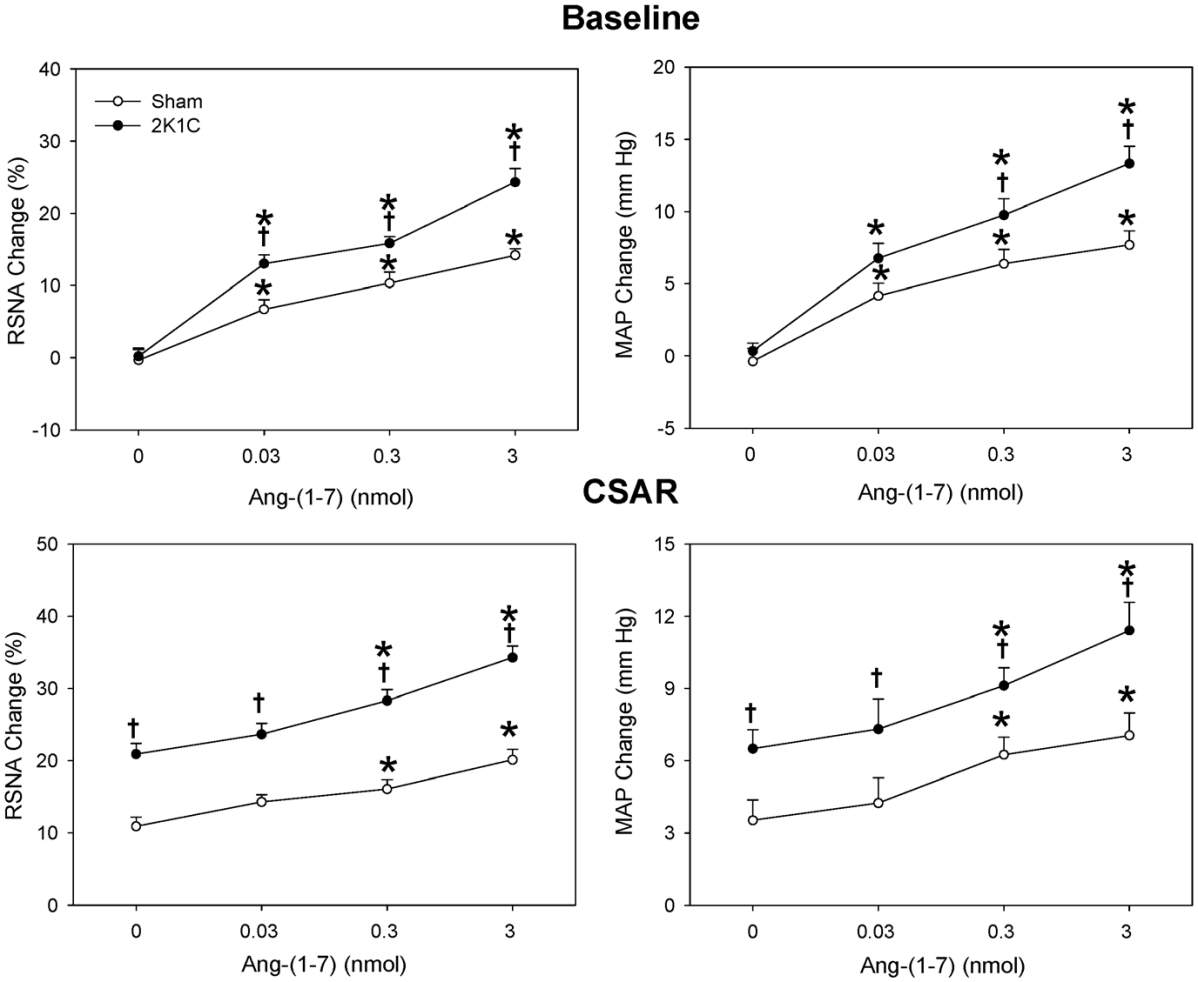
Effects of the PVN microinjection of saline and three doses of Ang-(1-7) (0.03, 0.3 and 3 nmol) on the baseline RSNA and MAP and CSAR. The CSAR was evaluated by the RSNA and MAP response to epicardial application of capsaicin (1 nmol). Values are mean ± SE. *P<0.05 compared with saline. † P<0.05 compared with Sham. n = 6 for each group.

The CSAR was enhanced in 2K1C rats compared with Sham rats which was consistent with our previous findings [3]–[5]. Bilateral PVN microinjection of either Ang II or Ang-(1-7) caused greater increases in baseline RSNA and MAP, and greater enhancement in CSAR in 2K1C rats than in Sham rats. Furthermore, there was no significant difference between the Ang II-induced and Ang-(1-7)-induced responses at the same dosage (Fig. 1). The increasing effects of Ang-(1-7) on RSNA and MAP were dose-related. Both middle and high dosages of Ang-(1-7) significantly enhanced the CSAR in 2K1C and Sham rats (Fig. 2).

### Effects of Ang II Combined with Ang-(1-7)

Simultaneous administration of Ang II and Ang-(1-7) in PVN caused much greater enhancing effects on baseline RSNA, MAP and CSAR than the same dosage of Ang II or Ang-(1-7) alone in 2K1C rats, but not in Sham rats (Fig. 1).

### Influence of Ang-(1-7) on the Effects of Ang II

**Figure 3:**
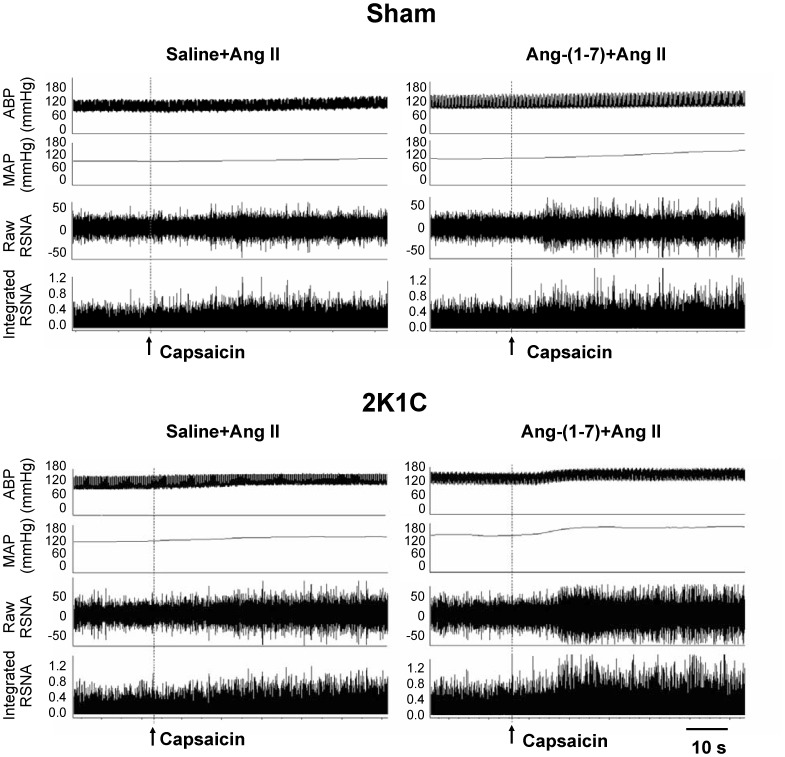
Representative recordings showing the effects of PVN pretreatment with saline or Ang-(1-7) (3 nmol) on the CSAR responses to the Ang II (0.3 nmol) in Sham and 2K1C rats. The CSAR was evaluated by the RSNA and MAP responses to epicardial application of capsaicin (1 nmol).

**Figure 4:**
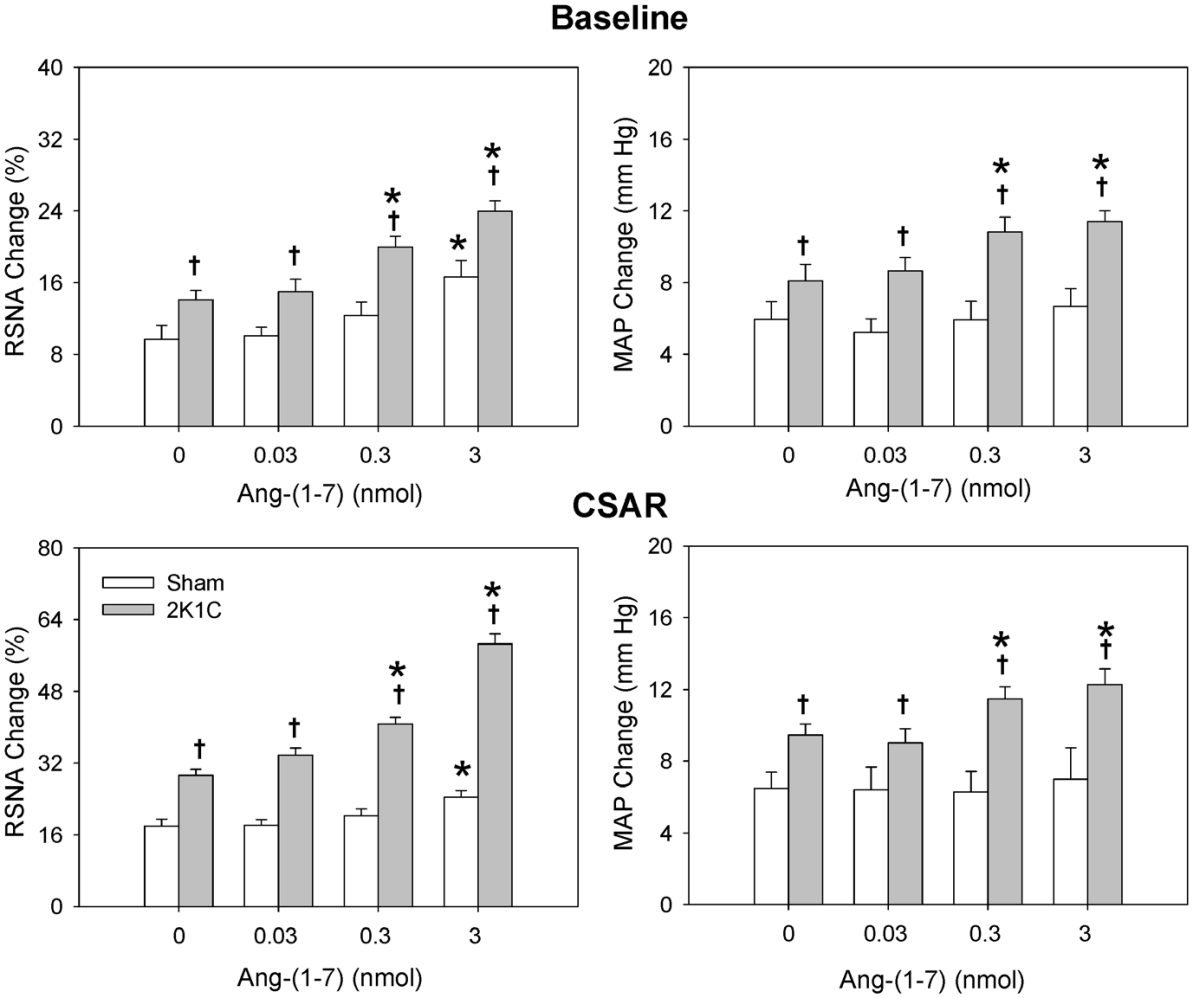
Effects of PVN pretreatment with saline and three doses of Ang-(1-7) (0.03, 0.3 and 3 nmol) on the baseline RSNA and MAP and CSAR responses to the Ang II (0.3 nmol). The CSAR was evaluated by the RSNA and MAP responses to epicardial application of capsaicin (1 nmol). Values are mean ± SE. *P<0.05 compared with saline. † P<0.05 compared with Sham. n = 6 for each group.

The representative recordings showed that the CSAR response to Ang II was enhanced in 2K1C rat compared with Sham rat and PVN pretreatment with high dose of Ang-(1-7) further significantly augmented the effect of Ang II on CSAR in 2K1C rat compared with saline (Fig. 3). PVN pretreatment with Ang-(1-7) augmented the effects of Ang II on baseline RSNA and MAP and CSAR in a dose-dependent manner in 2K1C rats, and both middle and high doses of Ang-(1-7) significantly elevated the effects of Ang II in 2K1C rats. However Ang-(1-7) had no significant influence on the RSNA, MAP and CSAR responses to Ang II in Sham rats (Fig. 4).

### Effects of Losartan

**Figure 5:**
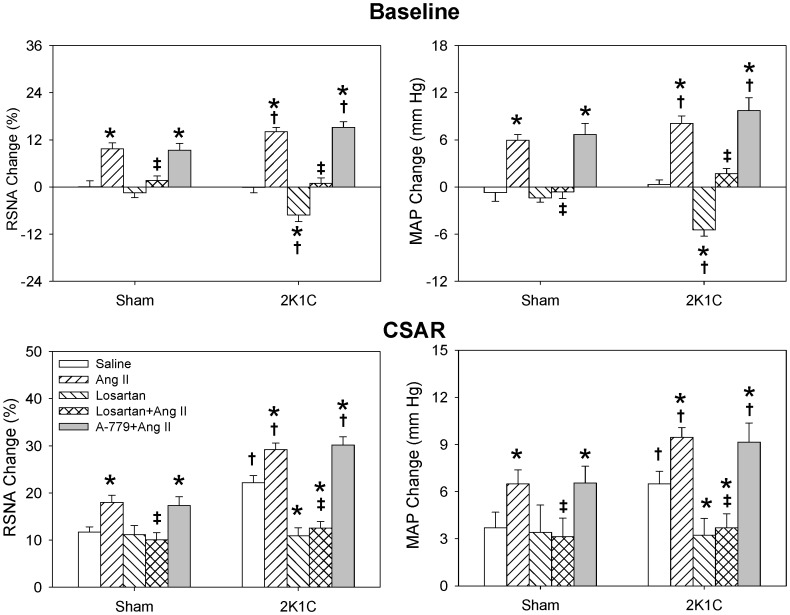
Effects of PVN microinjection of saline, Ang II (0.3 nmol), losartan (50 nmol), losartan+Ang II and A-779 (3 nmol) +Ang II on the baseline RSNA and MAP and CSAR. The CSAR was evaluated by the RSNA and MAP responses to epicardial application of capsaicin (1 nmol). Values are mean ± SE. * P<0.05 compared with saline. † P<0.05 compared with Sham. ‡ P<0.05 compared with Ang II alone. n = 6 for each group.

**Figure 6:**
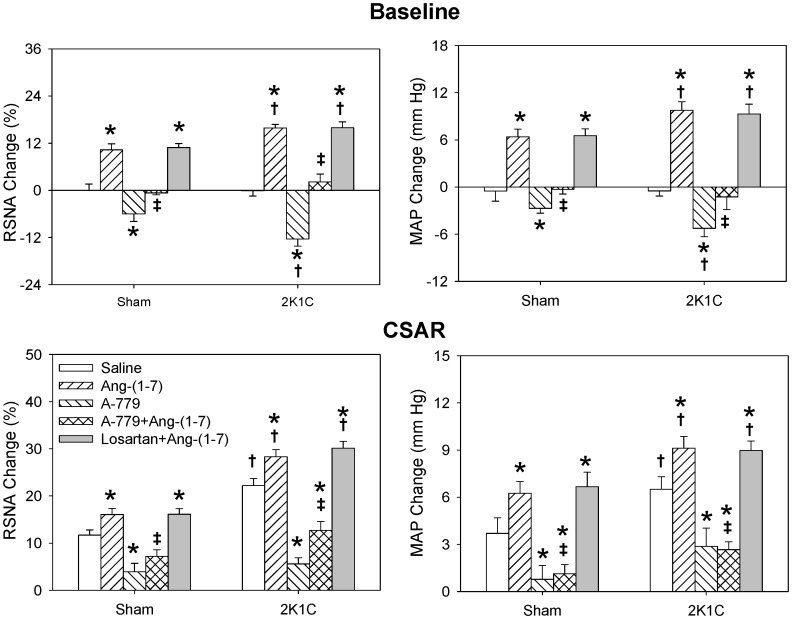
Effects of PVN microinjection of saline, Ang-(1-7) (0.3 nmol), A-779 (3 nmol), A-779+Ang-(1-7) and losartan (50 nmol) +Ang-(1-7) on the baseline RSNA and MAP and CSAR. The CSAR was evaluated by the RSNA and MAP responses to epicardial application of capsaicin (1 nmol). Values are mean ± SE. * P<0.05 compared with saline. † P<0.05 compared with Sham. ‡ P<0.05 compared with Ang-(1-7) alone. n = 6 for each group.

PVN microinjection with AT1 receptor antagonist losartan alone decreased baseline RSNA and MAP and normalized the enhanced CSAR in 2K1C rats but had no significant effect in the Sham rats which was consistent with our previous report [6]. Losartan in PVN abolished the effects of Ang II including increasing RSNA and MAP and enhancing CSAR in both 2K1C and Sham rats (Fig. 5), while it had no significant effect on Ang-(1-7) induced enhancement of RSNA, MAP and CSAR (Fig. 6).

### Effects of A-779

Mas receptor antagonist A-779 in PVN alone decreased baseline RSNA and MAP and attenuated the CSAR in both 2K1C and Sham rats, and the inhibitory effects in 2K1C rats were much greater than that in Sham rats. Pretreatment with A-779 in the PVN abolished the effects of Ang-(1-7) of increasing RSNA and MAP and enhancing CSAR in both Sham and 2K1C rats (Fig. 6), but had no significant effect on Ang II induced enhancement on RSNA, MAP and CSAR (Fig. 5).

### Ang II and Ang-(1-7) level, AT1 Receptor and Mas Receptor Protein Expression in the PVN

**Figure 7:**
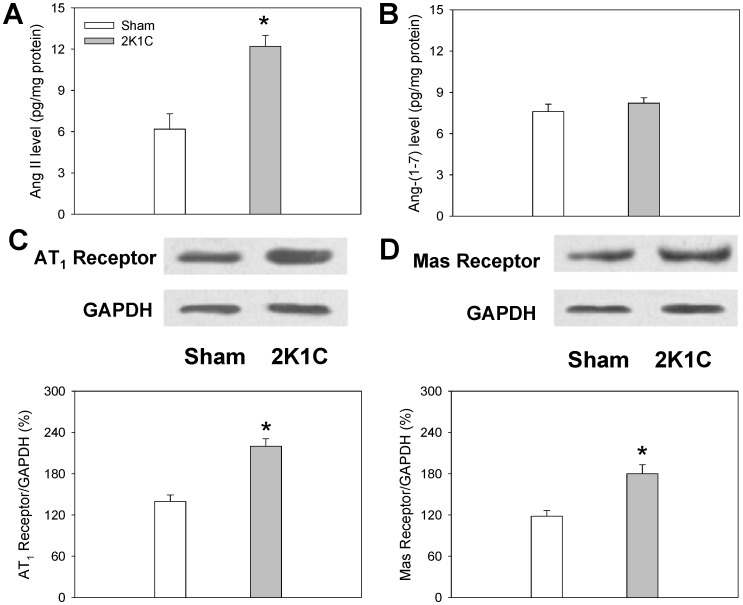
Ang II level (A), Ang-(1-7) level (B), AT_1_ receptor protein expression (C) and Mas receptor protein expression (D) in PVN in Sham rats and 2K1C rats. Values are mean ± SE. * P<0.05 compared with Sham rats. n  = 5 for each group.

Ang II level in PVN was increased in 2K1C rats but there was no significant difference in Ang-(1-7) level in the PVN between 2K1C and Sham rats. Both the Mas receptor and AT_1_ receptor protein expression in the PVN were significantly increased in 2K1C rats compared with Sham rats (Fig. 7).

## Discussion

Numerous studies have shown that sympathetic activity is enhanced in patients with essential [37], [38] or secondary hypertension [39], [40] and various hypertensive models [41]–[43], which contributes to the pathogenesis of hypertension and progression of organ damage [7]–[9]. CSAR is known as the sympathoexcitatory cardiovascular reflex which can be induced by stimulating cardiac sympathetic afferents and results in sympathetic activation [2]. In hypertension, the persistent increases in the load of left ventricle and myocardial oxygen consumption lead to myocardial hypertrophy and ischemia [44], [45]. Some released chemicals from ischemic myocardium, such as adenosine, bradykinin and hydrogen peroxide may stimulate cardiac sympathetic afferents and then enhance CSAR, in turn increase sympathetic output. Our previous studies have shown that the CSAR is enhanced in renovascular hypertensive rats and the enhanced CSAR partially contributes to the sympathetic activation and hypertension [3]–[5]. A recent study in our lab has shown that arterial baroreceptor and vagal afferents inhibit the CSAR and no significant difference in MAP and HR was found 1 hour after treatment with vagotomy and baroreceptor denervation compared with intact rats in both Sham and CHF rats. The CSAR is enhanced not only in CHF rats with bilateral vagotomy and arterial baroreceptor denervation, but also in intact CHF rats [32]. Vagotomy and baroreceptor denervation were carried out in the present study to minimize the confounding effect of the baroreflex on sympathetic activity and blood pressure. Therefore the present study was not performed under real physiological states, the inhibition of arterial baroreceptor and vagal afferents on CSAR should be considered if the CSAR is induced in intact animals. The primary new findings in the present study are that the Ang-(1-7) in PVN is as effective as Ang II in sensitizing the CSAR and increasing sympathetic outflow, and both endogenous Ang-(1-7) and Ang II in PVN contribute to the enhanced CSAR and sympathetic outflow in renovascular hypertension. Ang-(1-7) in PVN potentiates the effects of Ang II in renovascular hypertension. In contrast to Ang II, the effects of Ang-(1-7) are mediated by Mas receptors but not AT_1_ receptors.

The discovery of Ang-(1-7) and ACE2 adds a new cognition to the RAS family in the last decade. Ang-(1-7) and Ang II are now known as two important biological active peptides of RAS family. Studies have shown that Ang-(1-7) acts as a counter regulator of the Ang II effects [46], [47]. However, Ang-(1-7) microinjection into the caudal ventrolateral medulla (CVLM) caused similar depressor effects to Ang II in Wistar rats and spontaneously hypertensive rats (SHR) [48]; microinjection of Ang-(1-7) into the RVLM elicits a similar pressor response to Ang II in rats [49]–[51]. Chronic infusion of both Mas receptor antagonist A-779 and AT_1_ receptor antagonist losartan into the PVN prevent hypertension in a rat model of sleep apnea [28]. A recent study in our lab showed that Ang-(1-7) in the RVLM plays similar roles as Ang II in enhancing the CSAR and increasing the sympathetic outflow and blood pressure in normal rats [26]. Inhibition or lesion of the PVN abolishes the CSAR and the excitation of neurons in the PVN enhances the CSAR, suggesting that the PVN is an important component of the central neurocircuitry of the CSAR [13]. The RVLM receives projections from the PVN [14]. Both Ang II and Ang-(1-7) are known to be involved in the modulation of sympathetic drive and blood pressure in hypertension [50], [52]. The activity of Ang II and AT_1_ receptors in PVN contributes to the enhanced CSAR and sympathetic activation in renovascular hypertension [3], [6]. However, whether Ang-(1-7) in PVN is as effective as Ang II in modulating the enhanced CSAR and sympathetic outflow in renovascular hypertension is still unknown. In the present study, bilateral PVN microinjection of either Ang II or Ang-(1-7) caused greater increases in baseline RSNA and MAP, and greater enhancement in CSAR in 2K1C rats than in Sham rats. There was no significant difference between the Ang II-induced and Ang-(1-7)-induced responses of RSNA, MAP and CSAR at the same dosage. The increasing effects of Ang-(1-7) on RSNA and MAP were dose-related. Both middle and high doses of Ang-(1-7) significantly enhanced the CSAR in 2K1C and Sham rats. These results indicate that exogenous Ang-(1-7) in the PVN is as effective as Ang II in enhancing the CSAR and increasing sympathetic outflow in renovascular hypertension.

Our previous study has shown that simultaneous microinjection of Ang II and Ang-(1-7) into the RVLM causes greater effects than Ang II or Ang-(1-7) alone, which suggests a synergetic effect of Ang II and Ang-(1-7) in the RVLM in enhancing the CSAR and sympathetic outflow in normal rats [26]. In the present study, PVN microinjection of Ang II and Ang-(1-7) at the same time also caused greater enhancing effects on RSNA, MAP and CSAR than Ang II or Ang-(1-7) alone in 2K1C rats but not in Sham rats, suggested that there also exists a synergetic effect of Ang II and Ang-(1-7) in the PVN in enhancing the CSAR and sympathetic outflow in renovascular hypertensive rats.

It has been reported that pretreatment with Ang-(1-7) before Ang II enhances Ang II induced phosphorylation of ERK1/2 in mouse bone marrow-derived dendritic cells [27]. In the current study, PVN pretreatment with Ang-(1-7) augmented the effects of Ang II on RSNA, MAP and CSAR in a dose-dependent manner in 2K1C rats, both middle and high doses of Ang-(1-7) significantly enhanced the effects of Ang II in 2K1C rats. These results indicate that Ang-(1-7) in PVN potentiates the effects of Ang II on enhancing CSAR and sympathetic outflow in renovascular hypertension. Ang-(1-7) in PVN plays important roles in modulating the enhanced CSAR and sympathetic outflow in hypertension on the one hand by its own ability to increase RSNA, MAP and enhance CSAR; on the other hand via its potentiation of the Ang II effects. However the mechanisms responsible for the potentiation are unclear. Both the AT_1_ and the Mas receptor are G protein-coupled receptors [53]. We speculated that the mechanisms of this potentiation maybe attribute to the similar downstream signaling mechanisms of Ang II and Ang-(1-7) in the PVN in modulating RSNA, MAP and CSAR. Superoxide anions are known as an important Ang II intraneuronal signaling intermediate that leads to an increase in neuronal activity and elevated sympathetic output while nitric oxide has been shown to be a signaling molecule in Ang-(1-7) stimulated neurons that may counteract the signaling events of Ang II [54]. Our recent study indicate that superoxide anions mediate the effects of Ang II in PVN in 2K1C hypertension [3]. In the present study, the effects of Ang-(1-7) in the PVN on the RSNA, MAP and CSAR were similar to the effects of Ang II. It is known that nitric oxide in the PVN inhibits the Ang II-mediated increase in sympathetic nerve activity [55]–[58]. It would be impossible that nitric oxide mediate the enhancing effects of Ang-(1-7) in the PVN on the RSNA, MAP and CSAR. Recently, we found that superoxide anions but not nitric oxide in the RVLM are involved in modulating the enhanced CSAR, RSNA and MAP responses to Ang-(1-7) in the RVLM of rats [59]. Therefore we speculate that the excitatory effects of Ang-(1-7) in the PVN on the RSNA, MAP and CSAR may be also mediated by superoxide anions, which needs to be investigated in the future.

In the present study, we found that the effects of Ang-(1-7) in PVN, including increasing the RSNA and MAP and enhancing the CSAR, were blocked by Mas receptor antagonist A-779 but not AT_1_ receptor antagonist losartan in both 2K1C and Sham rats. The effects of Ang II in PVN were abolished by losartan but not A-779. These results suggest that the effect of Ang-(1-7) in the PVN is mediated by Mas receptors but not AT_1_ receptor, and the effect of Ang II in the PVN is mediated by AT_1_ receptor. Furthermore A-779 in PVN had no significant influence on the effects of Ang II in both 2K1C and Sham rats which indicate that endogenous Ang-(1-7) in PVN has no regulating roles in Ang II mediated effects.

Losartan in PVN alone decreased baseline RSNA and MAP and normalized the enhanced CSAR in 2K1C rats but had no significant effects in the Sham rats. While A-779 in PVN alone decreased baseline RSNA and MAP and attenuated the CSAR to a lower level than normal in both 2K1C and Sham rats, and the inhibitory effects in 2K1C rats were much greater than that in Sham rats. These results indicate that the activities of both endogenous Ang-(1-7)/Mas receptors and Ang II/AT_1_ receptors in PVN contribute to the enhanced CSAR, sympathetic outflow and high blood pressure in renovascular hypertensive rats. But Mas receptors rather than AT_1_ receptors in PVN are involved in the tonic control of sympathetic activity, blood pressure and CSAR in normal status; and Ang-(1-7) and Mas receptors may play more important roles in modulating enhanced CSAR and sympathetic output than Ang II and AT_1_ receptors in renovascular hypertension. However the mechanisms involved in the distinct contributes of Ang II/AT_1_ receptors and Ang-(1-7)/Mas receptors to CSAR and sympathetic output are still unclear and need to be studied in the future. In addition we found that Ang II level in the PVN was increased in 2K1C rats but there was no significant difference in Ang-(1-7) level in the PVN between 2K1C and Sham rats. Furthermore both the Mas receptor and AT_1_ receptor protein expression in the PVN were significantly increased in 2K1C rats compared with Sham rats. These results suggest that the roles of endogenous Ang II and AT_1_ receptors in modulating the RSNA, MAP and CSAR in 2K1C rats are resulted from both the increased production of Ang II and upregulation of AT_1_ receptors in the PVN, while the enhanced activity of endogenous Ang-(1-7) and Mas receptors in the PVN in 2K1C rats arises from the upregulation of Mas receptors in the PVN.

In conclusion, Ang-(1-7) in PVN is as effective as Ang II in enhancing the CSAR and increasing sympathetic outflow, and both endogenous Ang-(1-7) and Ang II in PVN contribute to the enhanced CSAR and sympathetic outflow in renovascular hypertension. Furthermore, Ang-(1-7) in PVN potentiates the effects of Ang II on the CSAR and sympathetic outflow in renovascular hypertension.
